# Single-nucleus transcriptomes reveal the underlying mechanisms of dynamic whitening in thermogenic adipose tissue in goats

**DOI:** 10.1186/s40104-025-01157-1

**Published:** 2025-02-09

**Authors:** Manman Li, Nange Ma, Shujie Luo, Yuyi Lu, Xixi Yan, Yang Li, Xinmei Li, Zhuohui Li, Zhipei Wu, Zhenyu Wei, Wei Wang, Huimei Fan, Yu Jiang, Yan Xiong, Yu Wang

**Affiliations:** 1 Key Laboratory of Animal Genetics, Breeding and Reproduction of Shaanxi Province, College of Animal Science and Technology, Northwest A&F University, Yangling, 712100 Shaanxi China; 2 Key Laboratory of Qinghai-Tibetan Plateau Animal Genetic Resource Reservation and Utilization, Ministry of Education, College of Animal and Veterinary Sciences, Southwest Minzu University, Chengdu, China; 3 Key Laboratory of Livestock Biology, Northwest A&F University, Yangling, 712100 Shaanxi China

**Keywords:** Beige adipocytes, snRNA-seq, Thermogenic adipose tissue, Whitening

## Abstract

**Background:**

Thermogenic adipose tissue, both beige and brown, experiences whitening as animals are exposed to warmth and age, but the potential mechanisms are not fully understood. In this study, we employed single-nucleus RNA-seq to construct a cell atlas during whitening progression and identified the characteristics of thermogenic adipocytes.

**Results:**

Our histological studies and bulk transcriptome gene expression analysis confirmed that both perirenal and omental adipose tissues (pAT and oAT) exhibited progressive whitening in goats. Compared to the classic brown adipocytes in mice, goat thermogenic adipocytes were more closely related in gene expression patterns to human beige adipocytes, which was also confirmed by adipocyte type- and lineage-specific marker expression analysis. Furthermore, trajectory analysis revealed beige- and white-like adipocytes deriving from a common origin, coexisting and undergoing the transdifferentiation. In addition, differences in gene expression profiles and cell communication patterns (e.g., FGF and CALCR signaling) between oAT and pAT suggested a lower thermogenic capacity of oAT than that of pAT.

**Conclusions:**

We constructed a cell atlas of goat pAT and oAT and descripted the characteristics of thermogenic adipocytes during whitening progression. Altogether, our results make a significant contribution to the molecular and cellular mechanisms behind the whitening of thermogenic adipocytes, and providing new insights into obesity prevention in humans and cold adaptation in animals.

**Supplementary Information:**

The online version contains supplementary material available at 10.1186/s40104-025-01157-1.

## Introduction

Brown adipose tissue (BAT) and beige (or brite) adipose tissue are thermogenic organs that expend energy by converting nutrient energy into heat, thereby protecting against hypothermia and obesity [1]. Thermogenesis, driven by the expression of uncoupling protein 1 (UCP1) in mitochondria, depends on uncoupling cellular respiration and mitochondrial ATP synthesis, which maintains body temperature of mammals in cold environments [2]. Nevertheless, thermogenic adipose tissues undergo a process of ‘whitening’ postnatally, resulting in white-like adipocytes recruitment.

The whitening of thermogenic adipocytes triggers not just morphological transformations-shifting from brown to white, expanding lipid droplets, a marked decrease in mitochondrial abundance, and adipocyte hypertrophy, but it also prompts changes in gene profiles. This includes the downregulation of genes associated with thermogenesis, the citrate cycle (TCA), and mitochondrial electron transport, alongside the upregulation of genes involved in metabolic processes such as fatty acid synthesis [3–5]. Although morphological assessments and traditional gene expression analyses can evaluate thermogenic adipocyte whitening, the dynamic changes in cell types and gene expression during the whitening of thermogenic fat at a single cell level remain unknown.

Thermogenic adipocytes of newborn rodents and primates are fully recruited prenatally in the interscapular BAT [6]. In human faints, BAT is located in supraclavicular and rapidly transform into white adipocytes in adult humans [7]. Cold recruits BAT mostly in young adults [8], even in middle-aged and aged people [9]. Rodent model organisms possess immature BAT at birth, which matures postnatally and is largely maintained throughout their lifespan because of housed at ~ 22 °C (room temperature) [2]. These models are extensively studied to understand the function and regulatory mechanism of thermogenic adipocytes. Compared to humans, interscapular BAT (iBAT) in mice could elevate its browning capacity and functionality stimulated by cold exposure and sympathetic activation, which is effective still in aged or high-fat diet mice [10], even mice kept under thermoneutral conditions [11, 12]. Recently, several studies have utilized single-cell RNA sequencing (scRNA-seq) to reveal new cell types, identify cell type markers, and track dynamic gene expression changes of specific cell types within mouse thermogenic fat in response to environmental stimulation such as cold stress, or thermoneutrality treatment [13–16].

Unlike rodents, larger ruminants are born with thermogenic adipose tissues that activate at birth and later undergo a whitening process similar to that in humans [17, 18]. Notably, 1-day-old muskox calves can tolerate ambient temperatures of −25 °C in the Arctic [19, 20], since deposits of highly thermogenic BAT around the heart, kidneys and mesentery produce the heat to maintain a constant body temperature. Previous studies have shown that the brown-like adipocytes in the perirenal adipose tissues of goats, sheep, and bovines transform dramatically into white-like adipocytes within weeks of birth [3, 5, 21]. It is noteworthy that perirenal and omental fat depots in newborn goat, sheep, bovine and muskox are thermogenic adipose tissues. Therefore, exploring visceral adipose tissues in newborn large ruminants, such as goat, would provide new insights and broaden our understanding of thermogenic fat roles, and are likely to mimic the whitening transition of humans.

Here, we generated a single-nucleus RNA sequencing (snRNA-seq) expression atlas of goat perirenal and greater omental adipose tissue (pAT and oAT) in the whitening process after birth. Our research revealed that these visceral adipose tissues (VAT) in newborn goats are predominantly composed of beige adipocytes. The whitening process represents the transdifferentiation from beige to white adipocytes, suggesting that goat VAT could serve as an effective model to study the whitening of thermogenic fat. Our study provides major insights into the whitening process of pAT and oAT, reveals potential markers for whitening, and serves as a foundational dataset to study how to treat metabolic diseases of humans and enhance the survival of young animals.

## Materials and methods

### Experimental animals

In this study, we collected samples that include pAT and oAT samples from male Guanzhong dairy goats (DG) at 3, 12, 20, 27-day old (D3, D12, D20, D27) and 1-month-old (M1) postnatal from Shanxi during early spring, as well as pAT samples from male Jianzhou big-eared goats (BEG) at 2-day-old (D2) and 1-year-old (Y1) postnatal from Sichuan during summer. The goats were purchased from local breeding farms. For each group, samples isolated from at least three goats were used for histological analysis and sequencing library construction. All experimental procedures involved in this study were approved by the Experimental Animal Manage Committee of Northwest A & F University.

### Histology analysis

The pAT and oAT were fixed by 4% paraformaldehyde (PFA) (Servicebio, Wuhan, China) and dehydrated, then embedded in paraffin and cut into sections. Immunohistochemistry staining was performed to examine the change of the UCP1 expression levels. For Immunohistochemistry staining, the tissue sections were performed with deparaffinization and rehydration, then treated in sodium citrate buffer and heated for antigen retrieval. Next, H_2_O_2_ were used to block the endogenous peroxidase activity, and then the sections were incubated with UCP1 primary antibodies (1:500, ab10983, Abcam) [22–25] at 4 °C overnight. Parallel controls were run with phosphate-buffered saline (PBS). Then, the sections were washed with PBS and incubated with HRP-linked secondary antibodies at room temperature for 2 h. After washing three times, the sections were visualized with diaminobenzidine (DAB) staining. Counterstaining was performed with hematoxylin and the slides were sealed with resins ready to image. Hematoxylin and eosin (H&E) staining was performed to characterize the tissue morphology. For H&E staining, rehydrated paraffin sections were stained with hematoxylin solution and then washed for 3 times. The sections were rinsed with 1% HCl (v/v) ethanol solution and dehydrated followed by washing with water for 5 min. Finally, the sections were stained with 1% eosin ethanol solution, mounted with resins, and covered with coverslips.

### Quantification of the brown adipocyte content

We used the web tool BATLAS to estimate the percentage of brown adipocytes in complex adipose tissue samples using RNA-seq data by combining gene signatures common between mice and human [26]. Firstly, we prepared uploaded file and ensured no duplicate genes and samples in RNA-seq data. The uploaded file must contain the expression of 98 brown marker genes and 21 white marker genes in the author’s manuscript [26]. Then, we set the expression of missing genes as zero and uploaded the prepared file at the web tool BATLAS. Finally, we obtained the percentage of brown adipocytes in each adipose tissue sample.

### Tissue dissociation and preparation of single-nucleus suspensions

Fresh isolated tissues were immediately placed in ice-cold DMEM (Gibco, Grand Island, USA) supplemented with 1% fetal bovine serum (FBS, Gibco, USA) to preserve viability. Fat tissues were washed 2–3 times with PBS, cleaned from blood and connective tissue and dissected into smaller pieces on ice. The tissue fragments were then collected in sterile tube of gradient cooling box and stored at −80 °C for isolation of nuclei. Frozen goat VAT were thawed on ice, minced to 1 mm^3^ and the nuclei were extracted using Nuclei EZ Prep (NUC101-1KT, Sigma-Aldrich). The minced VAT was filtered using a 40-μm nylon cell strainer and centrifuged at 500 × *g* for 5 min at 4 °C to obtain the pellet. The pellet was resuspended in PBS with 0.04% BSA and kept on ice for further testing. Trypan blue staining and microscopic examination indicated that the sample fragment rate was lower than 20% and the aggregation rate was lower than 10%. The number of nuclei was enumerated by microscopy, with an average yield of 400,000 nuclei per sample. Additionally, the RNA integrity number of the sample must exceed 7 after tissue processing.

### Single-nucleus RNA sequencing (snRNA-seq)

Adjust the single-nucleus suspension to an appropriate concentration for sequencing (700–1200 nuclei/μL), with a loading quantity of 25,000 nuclei per sample. Single-cell suspensions were converted to 10X-based snRNA-seq libraries according to standard protocols of the Chromium single-cell V3.0 reagent kit. All the remaining procedures were performed according to the user guide of Chromium Next GEM Single Cell 3ʹ Reagent Kits released by 10X genomics (10X Genomics, USA). Sequencing of libraries were performed by a NovaSeq (Illumina). The targeted sequencing depth is on average over 20,000 reads per cell.

### Processing of snRNA-seq data

Raw data were demultiplexed by 10X Genomics Cell Ranger software (version 5.0). The goat genome assembly ARS1.2 were used for mapping. The output FASTQ files were then imported to Seurat version 4.4.0 for the following analyses [27]. First, we applied DoubleFinder package to exclude potential doublets [28]. Nuclei with UMIs (≥ 10,000), mitochondrial genes (≥ 5%), ≤ 200 detected genes, or ≥ 3,500 detected genes were excluded. The qualified cells were filtered and normalized according to the developer’s vignettes. The batch effect was corrected using a canonical correlation analysis (CCA) from the Seurat package, then the uniform manifold approximation and projection (UMAP) and cluster analysis were performed to visualize the data in a two-dimensional space. Marker genes for cell type identification were found by utilizing the “FindAllMarkers” function and in previous reports. Differentially expressed genes (DEGs) between different groups were identified by “FindMarkers” function. Kyoto Encyclopedia of Genes and Genomes (KEGG) pathways enrichment analysis was conducted by the KEGG Orthology-Based Annotation System (KOBAS) (v. 3.0) [29].

### Pseudotime analysis

Pseudotime trajectories of adipocyte-progenitors, preadipocytes, and adipocytes were analyzed using a cell-gene matrix by Monocle (Version 2.18.0) [30], which reduced the space to two dimensions and ordered the cells. The “estimateSizeFactors” and “estimateDispersions” functions extracted and normalized the raw counts of cells. The “differentialGeneTest” function determined the variable genes with a model parameter “fullModelFormulaStr”. Subsequently, the trajectory was visualized in the reduced dimensional space, using “reduceDimension” and “orderCells” functions. Additionally, monocle developed “BEAM” function to analyze branch-dependent gene expression, the “plot_genes_branched_heatmap” function generated the heatmap showing the expression patterns.

### Cell–cell communication analysis

Extract the normalized data required by CellChat [31] from the Seurat object and create individual CellChat objects for pAT and oAT. Based on the UMI count matrix of each group, CellChat objects were created. Subsequently, import the ligand-receptor database into each CellChat object, pre-process the expression data, and infer the cell–cell interactions. Cell–cell communication analysis was then performed via the default setting with the database of ‘‘CellChatDB.human’’. By using the mergeCellChat function, the comparison of the total number of interactions and interaction strength was obtained by merging the CellChat objects of each group. The netVisual_diffInteraction function was employed to visualize the differential number of interactions or interaction strength among different cell populations. Finally, the rankNet function identified differentially expressed signaling pathways, and the plotGeneExpression function was used to visualize the distribution of signaling gene expression between different datasets.

### Bulk RNA-seq analysis

Total RNA was extracted from pAT and oAT of goats using TRIzol (Invitrogen, CA, USA). After quality control and quantification of the RNA, sequencing libraries were generated using NEBNext Ultra RNA Library Prep Kit from Illumina according to the manufacturer’s recommendations. The libraries were sequenced on an Illumina Hiseq 4000 platform and 100 bp/150 bp paired-end reads were generated.

Raw reads were filtered by Trimmomatic (v.0.33) [32]. Genomic alignment was performed using HISAT2 (version2.0.5) aligner to goat reference genome (ARS 1.2) [33]. The alignment results were sorted and merged by SAMtools (v. 0.1.19) [34]. The gene expression levels were quantified using StringTie (v. 1.2.2) [35], which employed transcript annotation to determine the quantification (in fragments per kilobase of transcript per million mapped reads, FPKM). Finally, DEGs between groups were identified through DESeq2 (v. 1.20.0) with |log_2_(fold change)| > 1 and *P*-value < 0.05 as thresholds [36].

### Statistical analysis

GraphPad prism 8.0 software was used to analyze the data. Multiple comparisons were performed using a one-way ANOVA. *P* < 0.05 represents significance. Data are presented as the mean ± standard error.

## Results

### Progressive whitening of goat thermogenic fat after birth

**Fig. 1:**
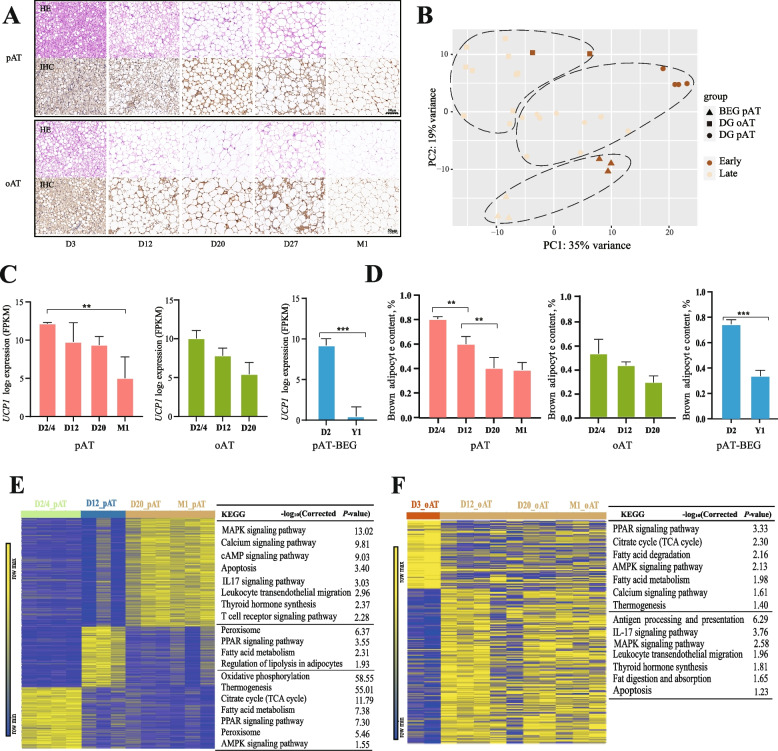
Histological and transcriptional remodeling during whitening of the pAT and oAT in goats. **A** Representative H&E and UCP1 immunohistochemical stained images of adipose tissue depots from dairy goats at different ages (*n* = 3). Scale bars represent 50 μm. “D” refers to “day old” and “M” refers to “month old”. **B** Principal component analysis (PCA) plot of pAT and oAT at different ages measured by using bulk RNA-seq. **C** Average log2 expression level of *UCP1* in goat pAT and oAT at the indicated age from bulk RNA-seq. FPKM, fragments per kilobase of transcript per million mapped reads. **D** The brown adipocyte content of pAT and oAT at different ages was calculated by the BATLAS web tool. **E** and **F** Heatmaps displaying k-means clustering of differential expression genes (DEGs) in pAT (**E**) and oAT (**F**) of dairy goats (left panel). The pathways enriched for each cluster were labeled in the right panel. **P < 0.05, ***P < 0.01 by one-way ANOVA followed by Bonferroni’s multiple comparisons

To characterize thermogenic fat whitening in goats, we collected pAT and oAT samples from Guanzhong DG at D3, D12, D20, D27, and M1 postnatal from Shannxi during early spring (February with an average temperature of ~ 10 °C), as well as pAT samples from Jianzhou BEG at 2-day-old and 1-year-old (D2 and Y1) postnatal from Sichuan during summer (August with an average temperature of ~ 30 °C). All depots had the typical morphology of thermogenic fat cells in neonatal goats, and the adipose tissue whitening, indicated by adipocyte hypertrophy, and loss of UCP1 protein levels could be gradually observed over time after birth (Fig. 1A, Fig. S1A–C). To verify the morphological observations, we performed 31 bulk RNA-seq for VAT. Principal component analysis (PCA) revealed a distinct separation of the pAT and oAT samples from early (D3 pAT and oAT of DG, and D2 pAT of BEG) and late phase (D12, D20 and M1 pAT and oAT of DG, Y1 pAT of BEG), whereas the early phase of BEG pAT formed a cluster together with late phase of DG pAT, indicating that warmth could contribute to the thermogenic fat whitening (Fig. 1B). Consistent with immunohistochemistry results, the *UCP1* expression gradually declined in goat VAT and almost disappeared at Y1 of age (Fig. 1C). In addition, the brown adipocyte content also gradually declined (Fig. 1D), estimated by the BATLAS analysis [26]. These findings showed rapid whitening in postnatal goat thermogenic fat and suggested the fat depots, breeds, and environmental temperature effect on goat thermogenic fat whitening progression.

To further delineate the gene expression patterns of thermogenic VAT in goat at various ages, we employed K-means clustering on bulk RNA-seq profiles. The greatest alterations in gene expression were observed from phase D2/4 to D20 in pAT of DG, whereas oAT exhibited significantly DEGs from early phase D3 to D12 and fewer DEGs were screened among late phase D12, D20 and M1 (Fig. 1E and F, Fig. S1D). Pathway-enriched analysis showed that early upregulated DEGs in both pAT and oAT were involved in processes associated with BAT thermogenic activity, including fatty acid metabolism, thermogenesis, oxidative phosphorylation, AMPK signaling pathway, and TCA. Conversely, genes that changed later were related to processes such as the MAPK signaling pathway, calcium signaling pathway, focal adhesion, fat digestion and absorption, IL17 signaling pathway and leukocyte transendothelial migration, suggesting metabolic adaptation in response to the whitening of adipose tissue (Fig. 1E and F, Tables S1 and S2, Fig. S1D–F and Tables S3–5). In comparison with pAT and oAT, the DEGs in the former at each age point consistently exhibited thermogenic functions; while DEGs in the latter, except for D3, were predominantly enriched in processes correlating with the functions of white-like adipose tissues, such as fatty acid biosynthesis, extracellular matrix (ECM) formation, immune response, and inflammation (Fig. S1F and Table S5). The aforementioned pathway analysis was confirmed in the pAT from BEG (Fig. S1G and Table S6). These results demonstrate that postnatally continuous whitening of the brown-like adipocytes into the white-like adipocytes and the rate of whitening in oAT was observed to be faster than that of pAT. Furthermore, we did not observe enriched of apoptotic pathway in all the enrichment analysis mentioned above (Fig. 1E and F, Fig. S1D–G and Tables S1–6), implying that the thermogenic fat whitening in goat is not caused by thermogenic adipocytes apoptosis.

### Single-nucleus transcriptome maps the whitening cellular atlas of goat thermogenic fat

**Fig. 2:**
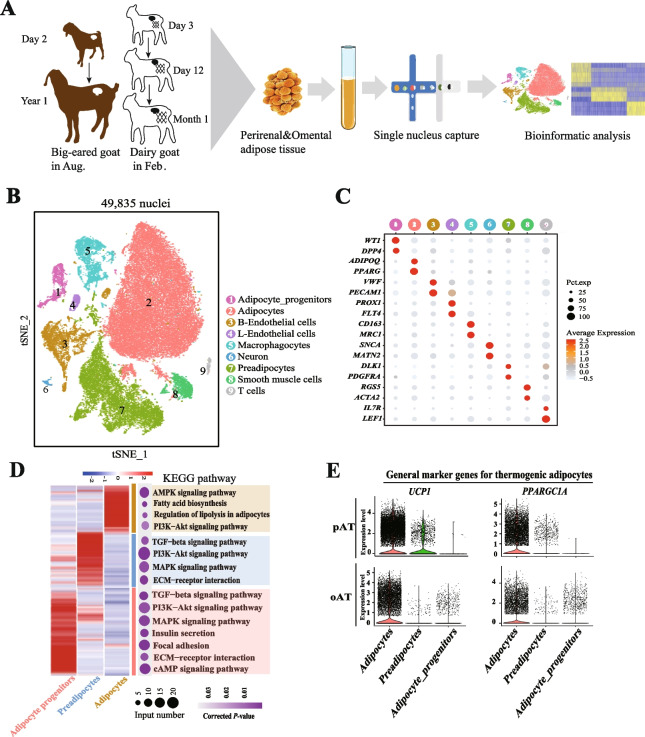
Single-nucleus transcriptomic atlas of pAT and oAT whitening in goats. **A** Schematic of the single-nucleus transcriptome experimental workflow in goat therogenic fat. **B** UMAP visualization of 9 cell types embedding 49,835 nuclei using snRNA-seq. **C** Dot plot showing the RNA expression levels of representative marker genes for 9 cell types. **D** Heatmap for the RNA expression levels of the top 200 marker genes in 3 cell types (left panel). Dot plot showing the pathway enrichment of DEGs for each cell type (right panel). **E** Expression levels of thermogenic adipocytes-associated general marker genes (*UCP1* and *PPARGC1A*) in adipocytes, preadipocytes, and adipocyte-progenitors of pAT and oAT measured by snRNA-seq

To investigate the molecular characteristics and alterations during thermogenic fat whitening at the single-cell level, we performed snRNA-seq on eight samples that respectively include three pAT and three oAT samples from DG at D3, D12, and ~ M1 postnatal, as well as two pAT samples from BEG at D2 and Y1 (Fig. 2A). A total of 49,835 nuclei were captured after removing empty droplets, outliers, cell debris, and potential doublets for further analysis. Unsupervised clustering and annotation by classical known cell-type-specific marker genes revealed 9 major cell types, including adipocyte-progenitors (*DPP4*^+^, *WT1*^+^), adipocytes (*PPARG*^+^, *ADIPOQ*^+^), preadipocytes (*PDGFRA*^+^, *DLK1*^+^), blood (B)-endothelial cells (*PECAM1*^+^, *VWF*^+^), lymphatic vessels (L)-endothelial cells (*PROX1*^+^, *FLT4*^+^), smooth muscle cells (*ACTA2*^+^, *RGS5*^+^), macrophagocytes (*MRC1*^+^, *CD163*^+^), T cells (*LEF1*^+^, *IL7R*^+^), and neuron (*MATN2*^+^, *SNCA*^+^) (Fig. 2B-C and Table S7, Fig. S2A).

Notably, the screened top 200 marker genes for each cell type were performed KEGG analysis, and the enriched pathways (setting the thresholds of corrected *P*-value < 0.05) were consistent with the cellular biological characteristics (Fig. 2D and Table S8, Fig. S2B and Table S9), confirmed the identification of cell types. Specifically, marker genes of adipocytes were enriched multiple metabolic related pathways, such as AMPK signaling pathway, fatty acid biosynthesis, regulation of lipolysis in adipocytes, and thermogenesis (corrected *P*-value = 0.074), implying the presence of subpopulations within adipocytes. Although both the pAT and oAT highly expressed general marker genes (*UCP1* and *PPARGC1A*) of thermogenic adipocytes (Fig. 2E), the expression level of *UCP1* was higher in preadipocytes of pAT than of oAT. The cellular atlas of goat fat was constructed, the differences of pAT and oAT were observed, and the characteristics of adipocytes in pAT and oAT needs to be further explored.

### Thermogenic adipocytes in goats possess characteristics of beige adipocytes

**Fig. 3:**
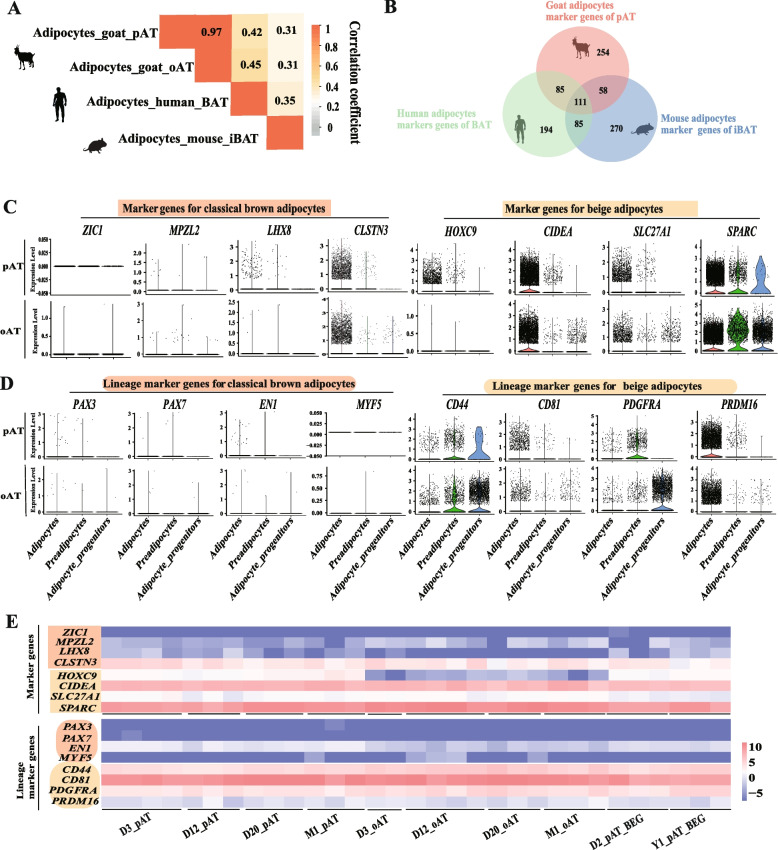
The characteristic identification of thermogenic adipocytes in goat VAT by brown or beige adipocytes marker genes profiling. **A** Spearman correlation coefficients between thermogenic adipocytes of goat, human, and mouse. The *P*-value of the Spearman correlation analyses are presented in Table S10. **B** Venn diagram illustrating overlapping marker genes (Top 5% expression levels) in goat, human, and mouse thermogenic adipocytes. **C** Expression levels of classical brown adipocytes-associated marker genes (*ZIC1*, *MPAL2*, *LHX8*, and *CLST3*) (left panel) and beige adipocytes-associated marker genes (*HOXC9*, *CIDEA*, *SLC27A1*, and *SPARC*) (right panel) in adipocytes, preadipocytes, and adipocyte-progenitors of pAT (left panel) and oAT (right panel) measured by snRNA-seq. **D** Expression levels of classical brown adipocytes-associated lineage marker genes (*PAX3*, *PAX7*, *EN1*, and *MYF5*) and beige adipocytes-associated lineage marker genes (*CD44*, *CD81*, *PDGFRA*, and *PRDM16*) in adipocytes, preadipocytes, and adipocyte- progenitors of pAT (top panel) and oAT (bottom panel) measured by snRNA-seq. **E** Heatmap illustrating gene expression levels of the aforementioned marker genes in (**C**) and (**D**) measured by bulk RNA-seq

To characterize the thermogenic adipocytes of goats, we first assessed the correlations between the adipocytes of goats and those of humans and mice as documented in previous studies [16, 37]. The adipocytes from pAT and oAT of goats had high correlation coefficient with the thermogenic adipocytes of human BAT, respectively (*r* = 0.42, *P* = 6.65E-209; *r* = 0.45, *P* = 2.98E-247), whereas they all had lower correlation coefficient with the adipocytes of mouse iBAT (*r* = 0.31, *P* = 1.51E-110; *r* = 0.31, *P* ≥ 2.25E-113) (Fig. 3A and Table S10). We also discovered a greater number of overlapping genes (top 5% expression levels) between goats and humans than those of goats and mice, including some important genes such as *ADIPOQ*, *EGFR*, *FGF2* and *PNPLA2* (Fig. 3B, Fig. S2C and Table S11). These results suggest that goat thermogenic adipocytes are more similar to human adipocytes from BAT than those of mice.

To deepen analysis of thermogenic adipocytes in goats, we examined the expression of four recognized brown and four beige adipocyte marker genes in goat thermogenic fats to address the characteristics of these thermogenic adipocytes. We observed higher expression of beige adipocytes marker genes (*HOXC9*, *CIDEA*, *SLC27A1*, and *SPARC*) compared to brown adipocytes marker genes (*ZIC1*, *MPZL2*, *LHX8*, and *CLSTN3*) in both pAT and oAT (Fig. 3C). Notably, the expression of homeobox genes *HOXC9*, a proposed beige marker, was absent in mouse iBAT and present in the retroperitoneal and inguinal subcutaneous depot [38]. Its expression was higher in pAT but not oAT (Fig. 3C), which may be due to the faster whitening of oAT. Conversely, *ZIC1* is a classical brown adipocyte maker in both mice and humans [6, 38, 39], and its expression was virtually absent in these two fat depots of goats (Fig. 3C). Previously, *SPARC* and *CLSTN3* served as markers of beige-like fat and BAT in adult human perirenal fat depot, respectively [40]. In our study, goat VAT had higher expression of *SPARC* compared to *CLSTN3* expression. These findings indicated goat thermogenic adipocytes are likely beige adipocytes.

To further explore these thermogenic adipocytes in goats derived from beige adipose developmental lineage, we analyzed the expression levels of four reported brown and four beige adipose tissue lineage markers in pAT and oAT at single-nucleus levels. The relative levels of classical brown adipocytes lineage marker genes (*PAX3*, *PAX7*, *EN1*, and *MYF5*) [41, 42], was virtually undetectable in both pAT and oAT. In contrast, the relative levels of beige adipocyte lineage marker genes (*CD44*, *CD81*, *PDGFRA*, and *PRDM16*) [17] was significantly enriched (Fig. 3D). Consistently, the bulk RNA-seq also revealed higher expression patterns of both aforementioned beige adipocyte and beige adipose developmental lineage marker genes than those of classical brown adipocyte markers in goat pAT and oAT (Fig. 3E). These results provide evidence that thermogenic adipocytes in goats exhibit a transcriptome compatible with beige adipocytes, including their ontogenic background.

### Evidences for beige- to white-like adipocytes transdifferentiation

**Fig. 4:**
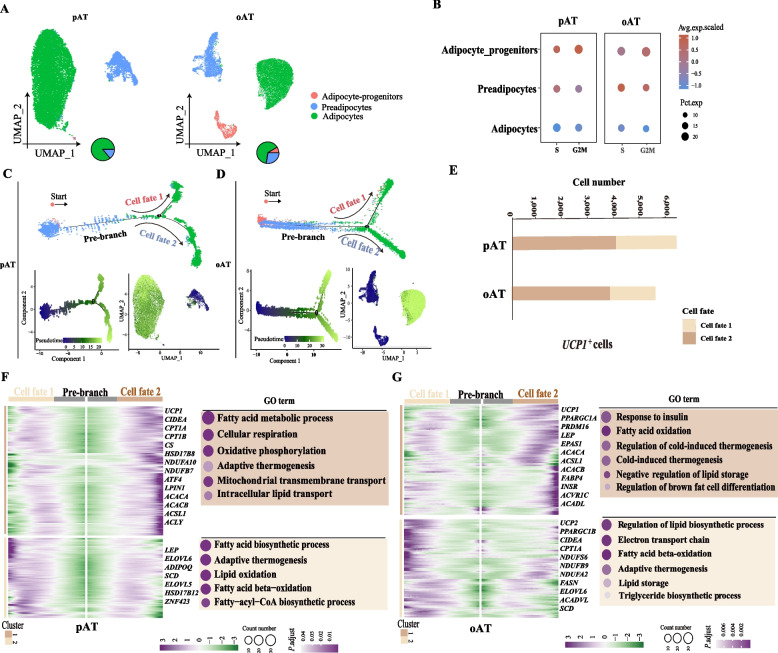
The origin and dynamics trajectories of goat beige adipocytes. **A** UMAP plots showing the adipocyte-progenitors, preadipocytes, and adipocytes in pAT (left panel) and oAT (right panel). Pie charts showing the percentage of the three cell types for pAT and oAT. **B** Dot plots showing expression of cell-cycle regulating genes in the three cell types. **C** and **D** Differentiation trajectories of the three cell types in pAT (**C**) and oAT (**D**). **E** The number of cells expressing *UCP1*(beige-like adipocyte marker) genes of cell fate 1 and 2 in pAT and oAT. **F** and **G** Heatmap illustrating the clustering and expression kinetics of DEGs for the pseudotime process of cell differentiation fate in adipocyte-progenitors, preadipocytes, and adipocytes in pAT (**F**) and oAT (**G**). The DEGs was clustered into 2 gene sets according to k-means. GO terms enriched for gene sets were labeled in the right panel

To explore the origins and dynamics trajectories of beige adipocytes during whitening, we focused on adipocyte-progenitors, preadipocytes, and adipocytes in pAT and oAT. The number of these cell types in pAT and oAT was shown (Fig. 4A). As expected, genes associated with the cell cycle exhibited higher expression levels in both the adipocyte-progenitors and preadipocytes, known for their robust proliferative capacity (Fig. 4B). Thus, we set adipocyte-progenitors as the initiating cell type and constructed differentiation trajectories by Monocle2 for these three cell types [43]. Two major branches, cell fate 1 and cell fate 2, were identified with a common differentiated pool of adipocyte-progenitors and preadipocytes in both pAT and oAT (Fig. 4C and D, Fig. S3). Further, a greater number of *UCP1*^+^ cells in cell fate 2 than that of cell fate 1 in both pAT and oAT was observed (Fig. 4E), which indicated adipocytes in cell fate 2 may possess more thermogenesis characteristics than that of cell fate 1.

To further verify the dynamic gene profiles of these two cell fates, the DEGs of two trajectory branches were screened. Adipocytes in cell fate 2 exhibited elevated expression of thermogenic genes (*UCP1*, *CIDEA*, *CPT1A*, *CS*, etc.) enriched in Gene Ontology (GO) terms related to “cellular respiration”, “oxidative phosphorylation”, and “cold-induced thermogenesis” (Fig. 4F and G, Tables S12 and S13). The adipocytes in cell fate 1 expressed higher levels of genes (*LEP*, *ELOVL6*, *ADIPOQ*, *SCD*, etc.) enriched for the GO terms, including “fatty acid biosynthetic process”, “regulation of lipid biosynthetic process”, and “electron transport chain” (Fig. 4F and G, Tables S12 and S13). These results indicated that the adipocytes in two cell fates were derived from a common origin and the coexistence of beige and white adipocyte characteristics during the whitening, which supported the occurrence of whitening through the transdifferentiation of beige- to white-like adipocytes in both pAT and oAT.

### Thermogenic oAT undergoes a more rapid rate of whitening than pAT

**Fig. 5:**
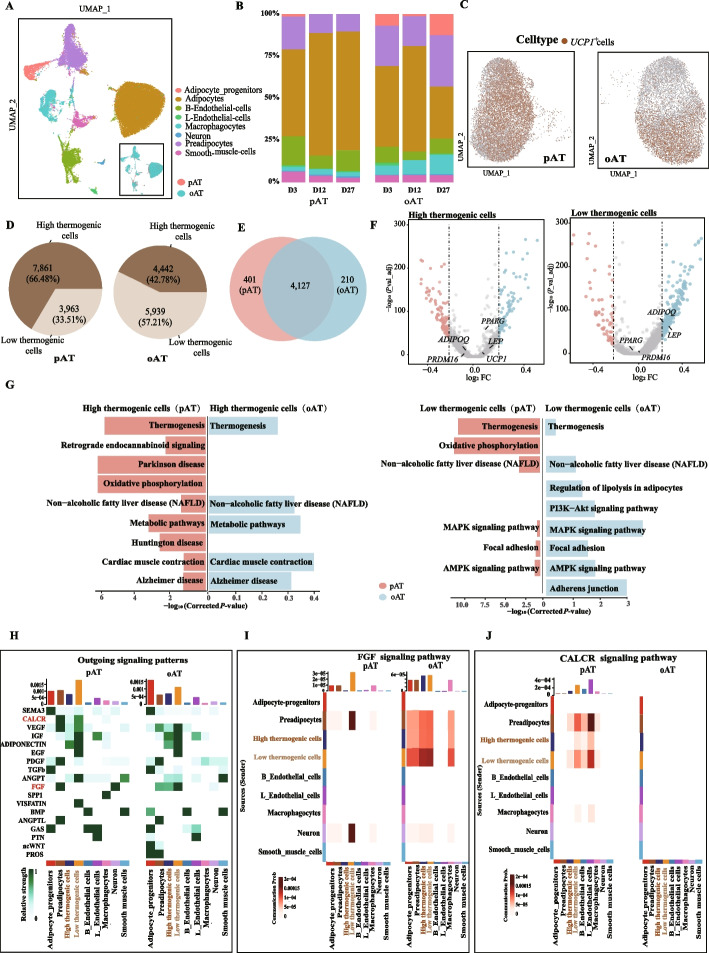
The differences of whitening progress between pAT and oAT. **A** UMAP visualization of 8 cell types and 2 tissues. **B** Bar plot showing the proportion of each cell type in pAT and oAT of dairy goats in 3 periods.**C** UMAP plots displaying the adipocyte cells in the pAT (left panel) and oAT (right panel). **D** Pie chart showing the proportion of adipocytes highly expressing (> 1) UCP1 or lowly expressing in the pAT (left panel) and oAT (right panel). **E** Venn diagram illustrating overlapping DEGs (between high thermogenic cells and low thermogenic cells) in pAT and oAT. **F** Volcano plot shows the DEGs in high thermogenic cells (left panel) and low thermogenic cells (right panel). **G** Enrichment analysis of DEGs of goat thermogenic cells in pAT and oAT. **H** Differences in outgoing signaling patterns of goat pAT and oAT. **I** and **J** Communication differences between FGF (**I**) and CALCR (**J**) signaling pathways in goat pAT and oAT

Currently, extensively reports regarded the oAT of humans and mice as a type of white adipose tissue [44]. However, our study showed that the oAT of newborn goats possessed thermogenic adipocytes. To clarify the potential differences between oAT and pAT in the progressive whitening process, we first compared the proportions of cell types in these two tissues (Fig. 5A). Relatively higher proportions of adipocyte-progenitors, preadipocytes, and macrophages in oAT (Fig. 5B), match with the features of oAT, including the composition of adipocyte progenitor-rich mesothelial sheets, innate immunological properties, and pronounced angiogenic activity adhering to adjacent structures [45, 46]. Then, we focused on adipocytes and according to expression levels of *UCP1*, identified those cells as high thermogenic cells (*UCP1* expression level ≥ 1) and low thermogenic cells (*UCP1* expression level < 1). In pAT, high thermogenic cells and low thermogenic cells were intermingled, whereas in oAT, high thermogenic cells and low thermogenic cells showed a more distinct clustering (Fig. 5C). In addition, we found relatively higher proportions (66.48% vs. 42.78%) of high thermogenic cells in pAT than oAT, whereas the percentage of low thermogenic cells was reverse (Fig. 5D), indicating lower thermogenic capacities of oAT than that of pAT.

Next, we compared the DEGs between high thermogenic cells and low thermogenic cells in two tissues, finding that most of the DEGs overlapped (91.14% in pAT and 95.16% in oAT), including genes such as *UCP1*, *PRDM16* and *PPARG,* implying that the overall thermogenic properties of the two tissues are similar (Fig. 5E, Table S14). To explore the differences, we further identified DEGs between pAT and oAT in two subpopulations, respectively (Fig. 5F). For high thermogenic cells, a total of 2,861 genes are highly expressed in pAT, and 1,794 genes are highly expressed in oAT. For low thermogenic cells, a total of 857 genes are highly expressed in pAT, and 3,185 genes are highly expressed in oAT (Tables S15 and S16). Additionally, in high thermogenic cells, there were no significant differences in the expression of key thermogenic markers (*UCP1, PRDM16*) and lipogenesis-related genes (*ADIPOQ, PPARG* and *LEP*) between pAT and oAT. In contrast, in the low thermogenic cells, *ADIPOQ* and *LEP* were significantly more expressed in oAT, while *PPARG* and *PRDM16* showed no significant differences. There are 7 overlapping DEGs that exhibit higher significance and a greater average log_2_(fold change) (> 0.5) in the expression levels of high thermogenic cells and low thermogenic cells, such as *TRPS1*, *SLIT2*, *TMEM132C*, *FGF2*, and *ZBTB16*, which might be the novel candidates for the regulation of the whitening of thermogenic fat. These findings also supported that whitening of oAT is faster than that of pAT, suggesting that, at the genetic level, oAT exhibits weaker thermogenic capacity but stronger lipogenic potential compared to pAT.

We then performed KEGG pathway enrichment analysis on DEGs between pAT and oAT and found that the thermogenic pathways enriched in both subpopulations were more prominent in pAT (Fig. 5G). Meanwhile, in oAT low thermogenic cells, the MAPK and AMPK pathways were significantly enriched, and specific enrichment of the PI3K-Akt pathway and adhesion pathways was observed (Fig. 5G, Tables S17 and S18). The enrichment results further emphasize the functional heterogeneity between the two tissues, with pAT having a stronger thermogenic capacity and oAT being more prone to whitening.

It's worth noting that the progress of VAT whitening is influenced by the microenvironment of adipose tissue. Therefore, we performed unbiased analyses of paracrine/autocrine signaling interactions using the cellchat [31], and found that low thermogenic cells in pAT or oAT exhibited stronger ECM and growth factor outgoing and incoming signaling interactions than high thermogenic cells, such as FGF, VEGF, IGF, and EGF signaling interactions (Fig. 5H, Fig. S4A). Among these ligands and receptors, FGF signaling and CALCR signaling showed a significantly differences between oAT and pAT adipocytes, respectively. In pAT, neither the high thermogenic cells nor the low thermogenic cells received the FGF signaling, with only the high thermogenic cells sending the FGF signaling. In oAT, both the high thermogenic cells and the low thermogenic cells received and sent the FGF signaling (Fig. 5I). This pattern was comparable to the thermogenic capacity of oAT and pAT adipocytes subpopulations and also consistent with previous reports that FGF2 can inhibit thermogenesis in brown and beige fat [47]. In pAT high thermogenic cells and low thermogenic cells send and received CALCR signaling, but not in oAT (Fig. 5J). Further studies are required to validate the roles of CALCR signaling in pAT. Additionally, the cellchat analysis of BEG pAT also confirmed these results (Fig. S4B–F). Altogether, these data from aspects of cell proportion, gene expression, pathway function and microenvironment indicated that goat oAT possess thermogenic capacity and undergoes a more rapid rate of whitening than pAT.

## Discussion

Age-related BAT whitening is not exclusive to human and has also been observed in animals, such as rabbits, bovines, sheep, and goats [3, 4, 21, 48]. As observed in previous studies, BAT rapidly disappears within 7–30 d after birth in goats and ovine [5, 49–51]. Here, we applied snRNA-seq to generate a comprehensive cellular atlas, determine in vivo differentiation trajectories, and study the plasticity of adipocytes subpopulations of pAT and oAT in goats. Our data revealed that the thermogenic adipocytes of goat VAT are likely beige adipocytes and whitening probably occurs through transdifferentiaion. Differences in gene expression and cell signals between oAT and pAT revealed the lower thermogenesis and more rapidly thermogenic adipocytes whitening in oAT*.* These findings provide valuable information for preventing whitening and potentially recruiting thermogenic adipocytes to combat metabolic diseases and enhance the survival of young animals.

Lineage tracing studies have showed that the two types (classical brown and beige) of thermogenic adipocytes stem from distinct developmental sources, exhibit overlapping yet different gene expression profiles [6, 39, 41, 42, 52]. Previous data suggest that classical brown fat derived from a MYF^+^ lineage, while the beige fat that emerge in white fat from a MYF5^−^ lineage. In our study, we found that thermogenic adipocytes in newborn goat adipocytes exhibited characteristics of beige adipocytes, as evidenced by the expression of lineage marker genes of beige adipocytes. Conversely, there was minimal expression of lineage marker genes of classical brown adipocytes, consistent with the results of classical maker genes for these two distinct types of thermogenic adipocytes. Thermogenic adipocyte tissue previously from the supraclavicular region of adult humans are composed of beige adipocytes [53]. Similarly, recent study also has showed that perirenal adipocyte in adult humans is similar to beige adipocyte, and has a higher capacity of browning compared to murine beige/brite adipose tissue [40]. Our results provided the evidence that goat thermogenic adipocytes possess the characteristics of beige adipocytes, and the neonatal goat thermogenic adipocytes could serve as a valuable model for exploring human thermogenic adipocytes. The existence of beige adipocytes in the larger mammals may represent an evolutionarily conserved cellular mechanism to provide flexibility in adaptive thermogenesis, where hypothermia is a less frequent threat than in rodents.

The analysis across all cell types in VAT allows us to explore the heterogeneity of thermogenic adipocytes and map the adipogenesis trajectory in vivo. Our results showed a lineage hierarchy in which adipocytes-progenitors were determined into preadipocytes, and subsequently differentiated into fully mature adipocytes, which was consistent with several works revealing adipogenesis in mice and humans [54–56]. In our Monocle analysis for cell fate 1 and 2 of adipocytes, apoptotic pathways were not identified. Instead, whitening occurs predominantly through the transdifferentiation of beige adipocytes into white-like adipocytes. It represents distinct phenotypic states resulting from adipocyte plasticity in response to environmental or individual cues. Consistently, thermogenic adipocytes can transdifferentiate into white adipocytes in mice [52], and rabbit BAT whitening is mainly driven by transformation of adipocyte fate rather than cell death [4]. In summary, we consider transdifferentiation of beige to white adipocytes is a significant component of the postnatal transformation of the perirenal and omental adipose depot in goat, using a time course with several time points.

Furthermore, the differential whitening speed of thermogenic adipose tissue in pAT and oAT of goat, gave reason to research the cellular components difference between these two depots. We discovered that oAT has a greater number of adipocyte-progenitors, preadipocytes, and immune cells, which is in line with its role in the body as the guardian of the abdomen [45]. Utilizing single-cell RNA sequencing and further analyses, we discovered a classical adipocyte subpopulation with high thermogenic cells coexisting with the low thermogenic cells within the beige adipose tissue of goat. Compared with the low thermogenic cells in pAT, these low thermogenic cells in oAT had substantially higher *ADIPOQ* and *LEP* expression. Functional analyses showed that, unlike the thermogenic beige adipocytes in oAT, the thermogenic beige adipocytes in pAT have higher thermogenesis and oxidative phosphorylation. The microenvironment analysis found low thermogenic cells in pAT and oAT exhibited stronger ECM and growth factor. These results suggested that the faster whitening progress of oAT compared to pAT was explained by perirenal fat surrounding the adrenal gland, which could secret epinephrine and norepinephrine upon sympathetic activation [57], to maintain perirenal fat thermogenic function in a paracrine way [40], thereby decelerating the whitening process of pAT. Thus, we propose that goats mimic the whitening progress observed in humans much more closely than mice that were utilized in prior studies.

## Conclusions

In conclusion, our research resolves the transcriptomic landscape associated with progressive whitening in goat visceral adipose tissue and offers insights into the cellular foundation of thermogenic adipose tissue whitening knowledge. Moreover, we emphasize that goat thermogenic adipocytes were more closely related to human beige adipocytes and undergone the whitening through transdifferentiation. Multiple analyses at the RNA level supported a lower thermogenic capacity of oAT than that of pAT. Consequently, goat visceral adipose tissue serves as an excellent model for investigating the mechanisms underlying the whitening of thermogenic adipose tissue in humans.

## Supplementary Information

Additional file 1: Table S1 The results of KEGG analyses of DEGs in dairy goats pAT (Fisher's exact test). Table S2 The results of KEGG analyses of DEGs in dairy goats oAT (Fisher's exact test). Table S3 The results of KEGG of DEGs in goats pAT and oAT. Table S4 The results of KEGG analyses of DEGs between any two samples. Table S5 The results of KEGG analyses of DEGs in pAT and oAT at every age (Fisher’s exact test). Table S6 The results of KEGG of DEGs in big-eared goat pAT. Table S7 The marker genes in cell types of goat adipose tissues. Table S8 The results of KEGG analyses of marker genes in adipocyte-progenitors, preadipocytes and adipocytes (Fisher's exact test). Table S9 The results of KEGG analyses of marker genes in blood (B)-endothelial cells, lymphatic vessels (L)- endothelial cells, smooth muscle cells, macrophagocytes, T cells and neuron (Fisher's exact test). Table S10 The *P*-value of spearman correlation analyses between thermogenic adipocytes of goat, human, and mouse. Table S11 The results of overlapping marker genes with the highest 5% expression levels of thermogenic adipocytes in goat, human, and mouse. Table S12 The results of GO analyses of the DEGs for the pseudotime process in pAT. Table S13 The results of GO analyses of the DEGs for the pseudotime process in oAT. Table S14 The results of overlapping marker genes of thermogenic adipocytes in pAT and oAT. Table S15 The DEGs of high thermogenic cells in pAT and oAT. Table S16 The DEGs of low thermogenic cells in pAT and oAT. Table S17 The results of KEGG analyses of the marker genes associated with high thermogenic cells in pAT and oAT. Table S18 The results of KEGG analyses of the marker genes associated with low thermogenic cells in pAT and oAT.

Additional file 2: Fig. S1 Transcriptional remodeling of the pAT and oAT in goats. (A and B) Representative pictures of goat pAT and oAT at different ages. (C) The number of adipocytes per region (*n*=5) in histological images of the pAT and oAT in goats. (D) Heatmap displaying k-means clustering of DEG in goats pAT and oAT. The pathways enriched for each cluster were labeled in the right panel. (E) Pathway analysis of DEG detected by comparing the any two samples. (F) Pathway analysis of DEG detected by comparing the pAT and oAT at every age. (G) Heatmap displaying k-means clustering of DEG in big-eared goat pAT. The pathways enriched for each cluster were labeled in the right panel. **P <0.05, ***P <0.01 by one-way ANOVA followed by Bonferroni's multiple comparisons. pAT, perirenal adipose tissue; oAT, omental adipose tissue.

Additional file 3: Fig. S2 Single-nucleus transcriptomic atlas of pAT and oAT in goats. (A) Feature plots showing the RNA expression levels of representative marker genes in 9 cell types of all 8 samples. (B) Heatmap for the RNA expression levels of the top 200 marker genes in 6 cell types (left panel). Dot plot showing the pathway enrichment of marker genes for each cell type (right panel). (C) Venn diagram illustrating overlapping marker genes with the highest 5% expression levels of thermogenic adipocytes in goat oAT, human BAT, and mouse iBAT. iBAT, interscapular BAT.

Additional file 4: Fig. S3 Differentiation trajectories of the three cell types in pAT and oAT. pAT, perirenal adipose tissue; oAT, omental adipose tissue.

Additional file 5: Fig. S4 The differential signaling in the cell-cell communication network analyzed by CellChat. (A) The incoming signaling patterns in pAT and oAT. (B) The snRNA-seq expression distribution of ligand and receptor in FGF signaling in pAT and oAT. (C and D) The outgoing (A) and incoming (B) signaling patterns in pAT and oAT. (E) The inferred FGF signaling network in D2 and Y1 pAT of big-eared goats. (F) The snRNA-seq expression distribution of ligand and receptor in FGF signaling in D2 and Y1 pAT of big-eared goats. pAT, perirenal adipose tissue; oAT, omental adipose tissue.

## Data Availability

The RNA-seq data obtained in this study have been deposited in the China National GeneBank (http://www.cngb.org) and assigned the project ID CNP000193 and CNP0006284.

## Abbreviations

BAT: Brown adipose tissue
BEG: Big-eared goat
BSA: Bovine serum albumin
CCA: Canonical correlation analysis
DAB: Diaminobenzidine
DEG: Differentially expressed gene
DG: Dairy goat
ECM: Extracellular matrix
FPKM: Fragments per kilobase of transcript per million mapped reads
GO: Gene Ontology
H&E: Hematoxylin and eosin
iBAT: Interscapular BAT
KEGG: Kyoto Encyclopedia of Genes and Genomes
oAT: Omental adipose tissue
pAT: Perirenal adipose tissue
PBS: Phosphate-buffered saline
PCA: Principal component analysis
PFA: Paraformaldehyde
PPARG: Peroxisome proliferator activated receptor-γ
scRNA-seq: Single-cell RNA sequencing
snRNA-seq: Single-nucleus RNA sequencing
TCA: The citrate cycle
UCP1: Uncoupling protein 1
VAT: Visceral adipose tissue
WAT: White adipose tissue
