# Supplementary figures and images for: Single-nucleus transcriptomes reveal the underlying mechanisms of dynamic whitening in thermogenic adipose tissue in goats
Source: J Anim Sci Biotechnol. 2025 Feb 9;16:23. doi: 10.1186/s40104-025-01157-1 (PMC11807308; doi:10.1186/s40104-025-01157-1)

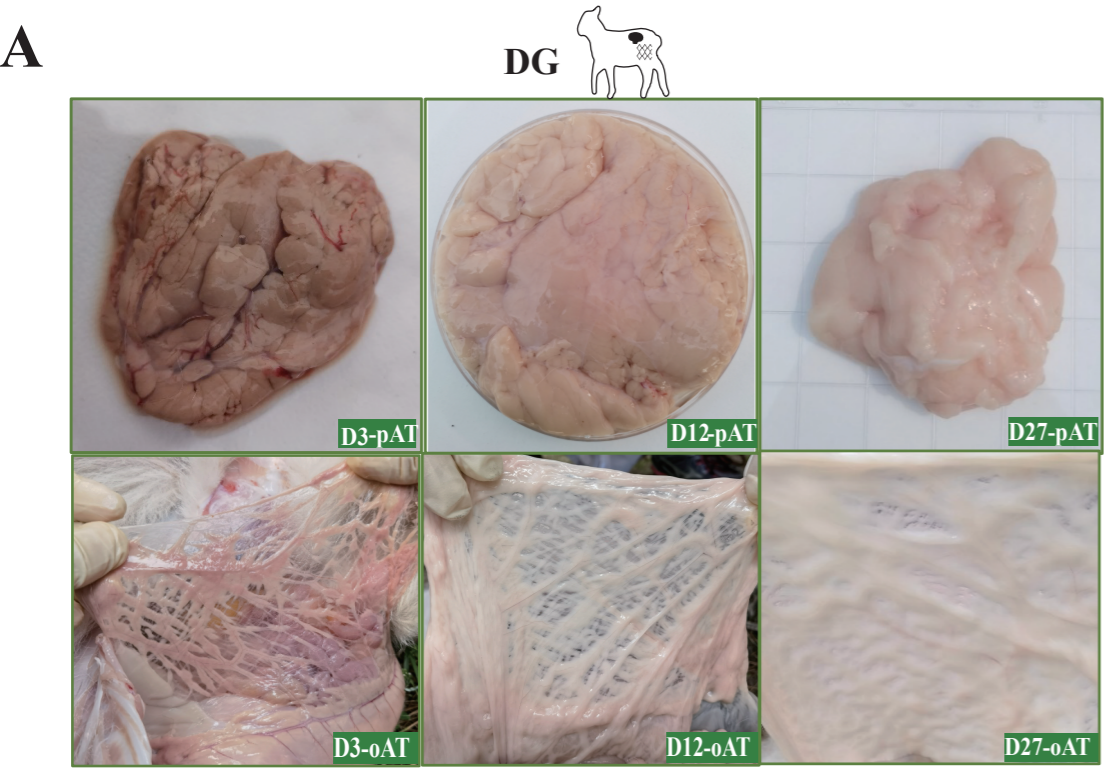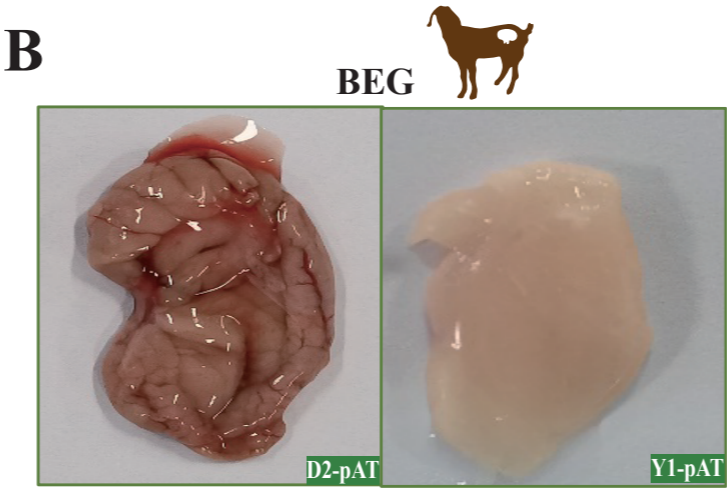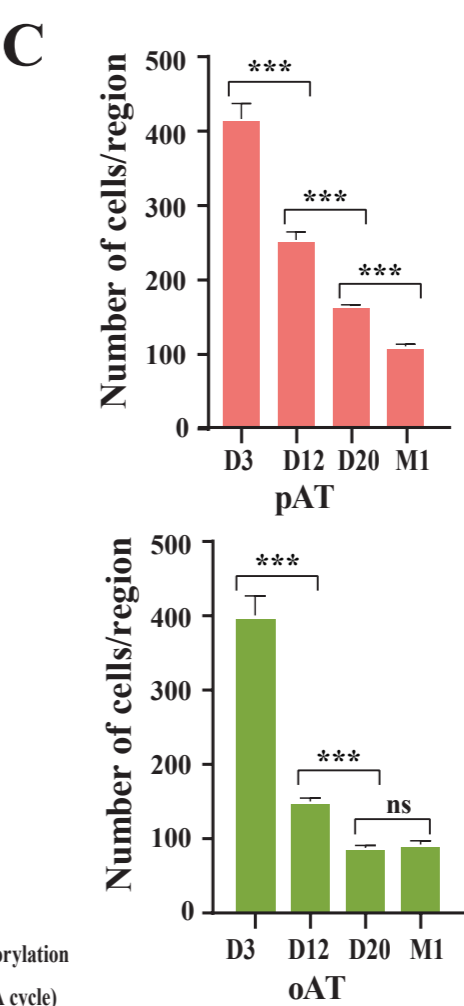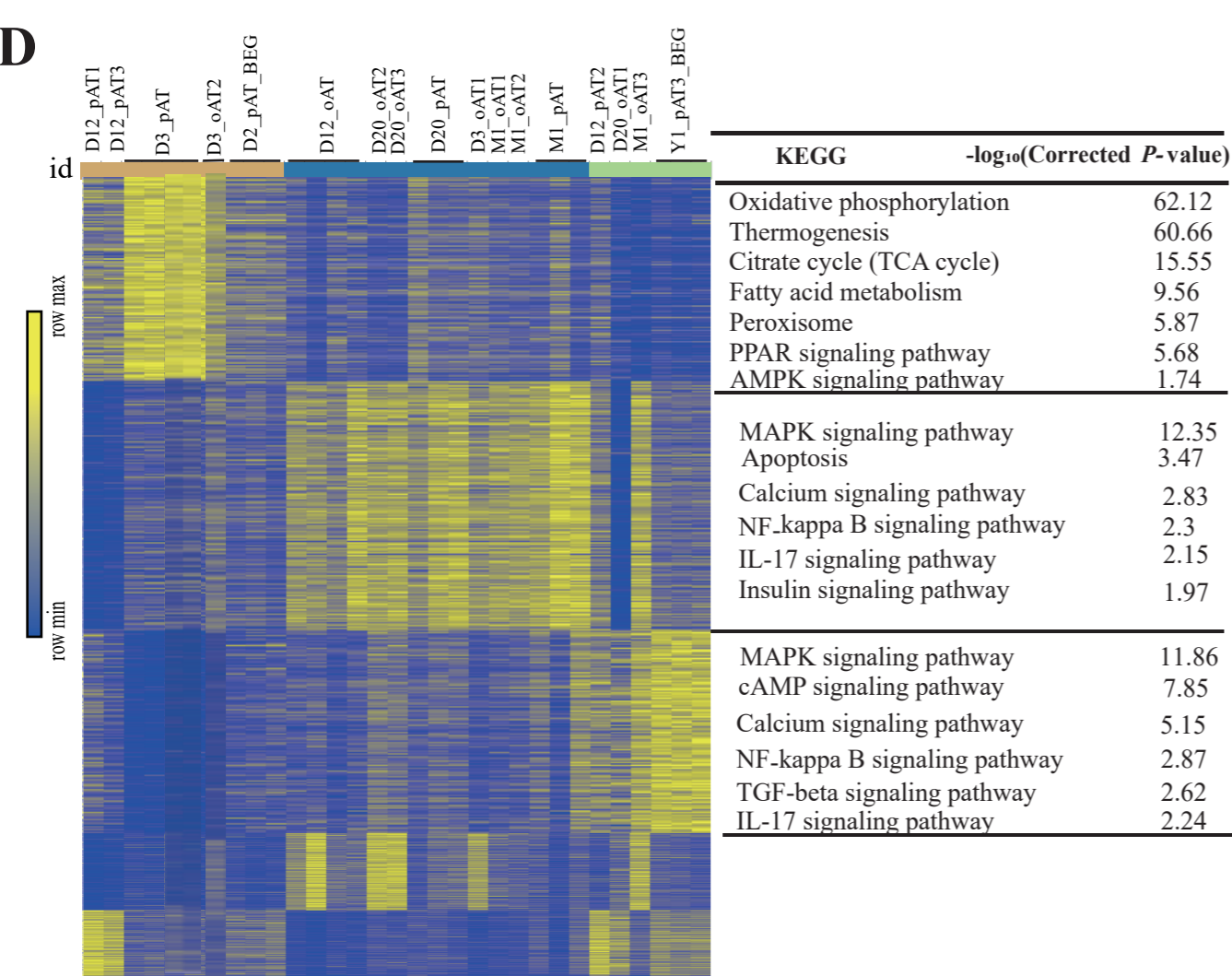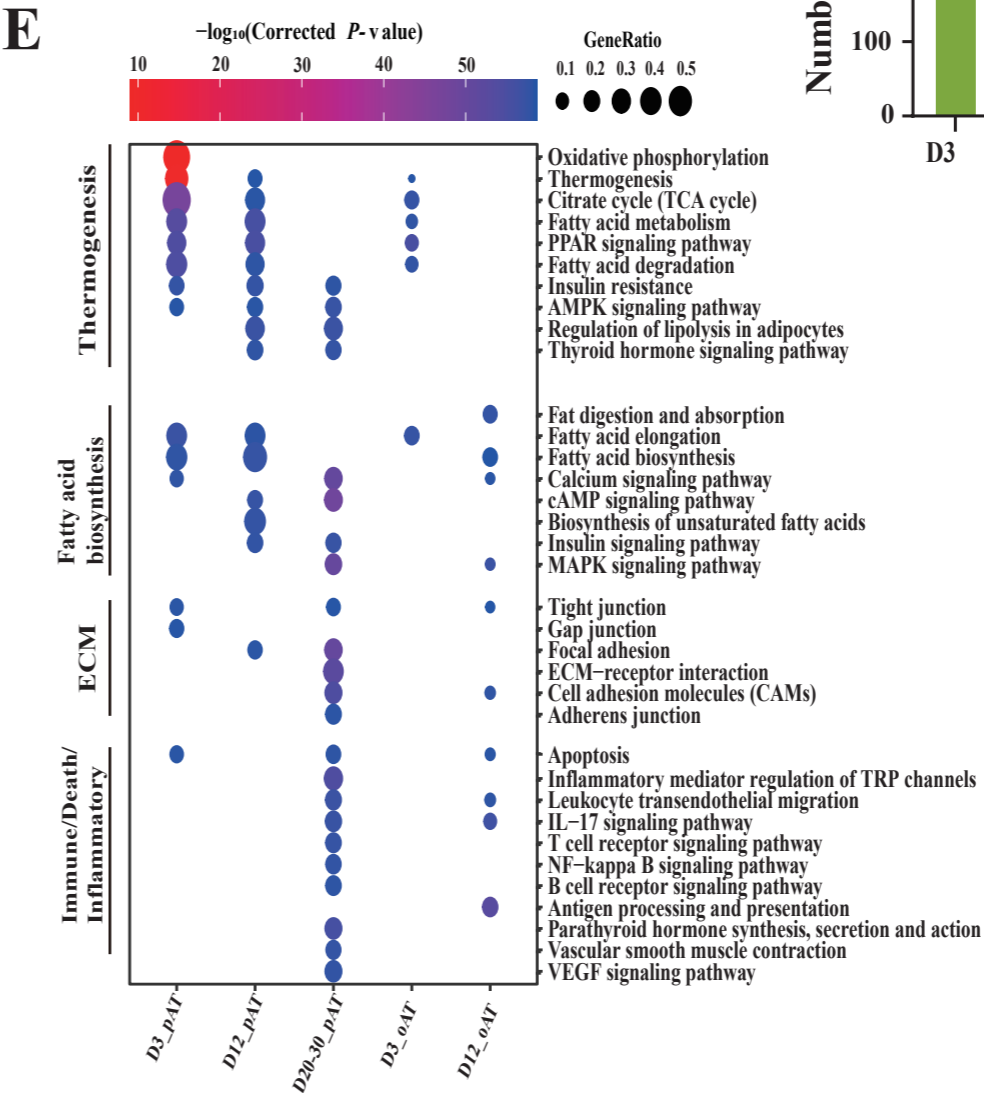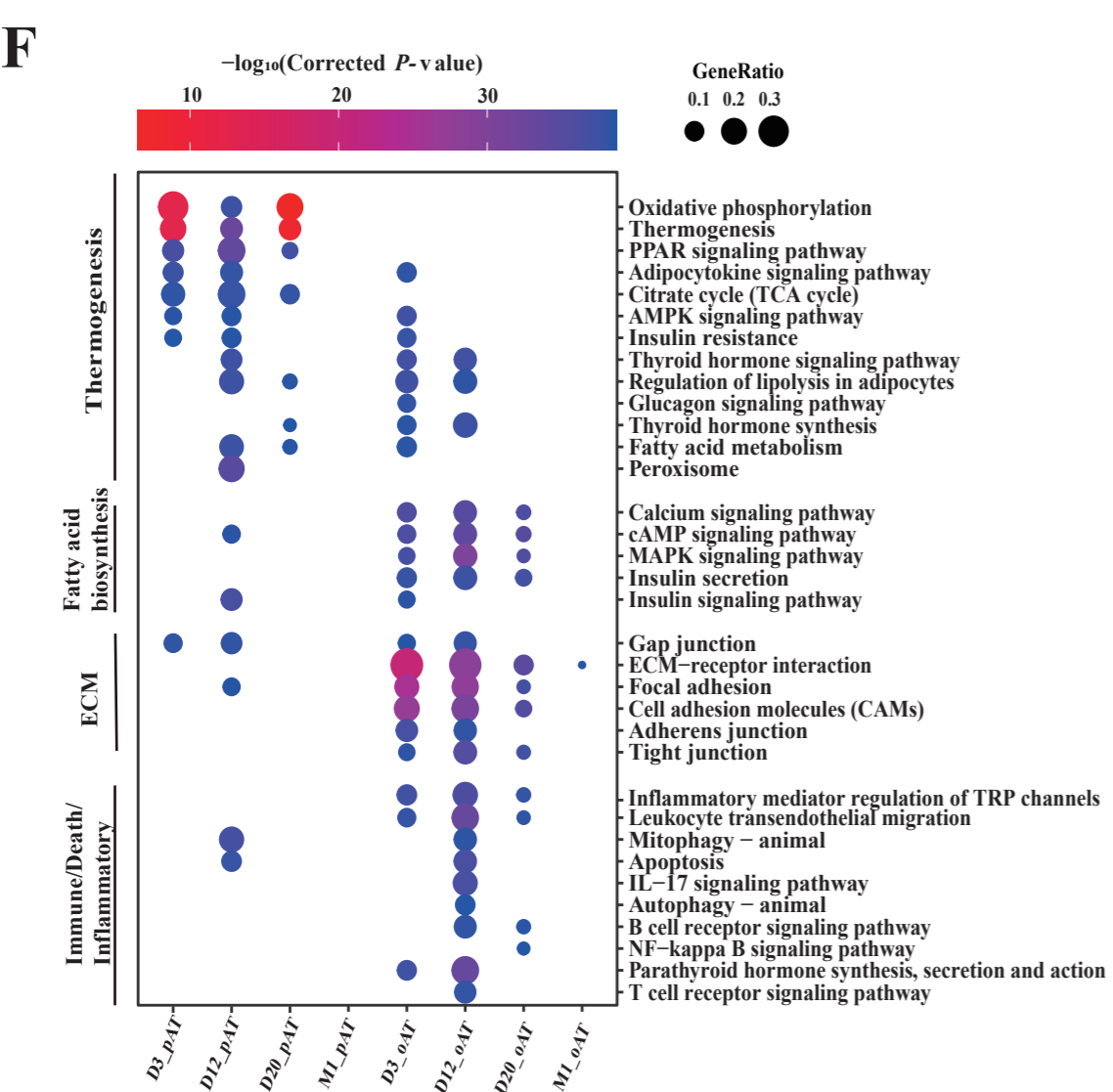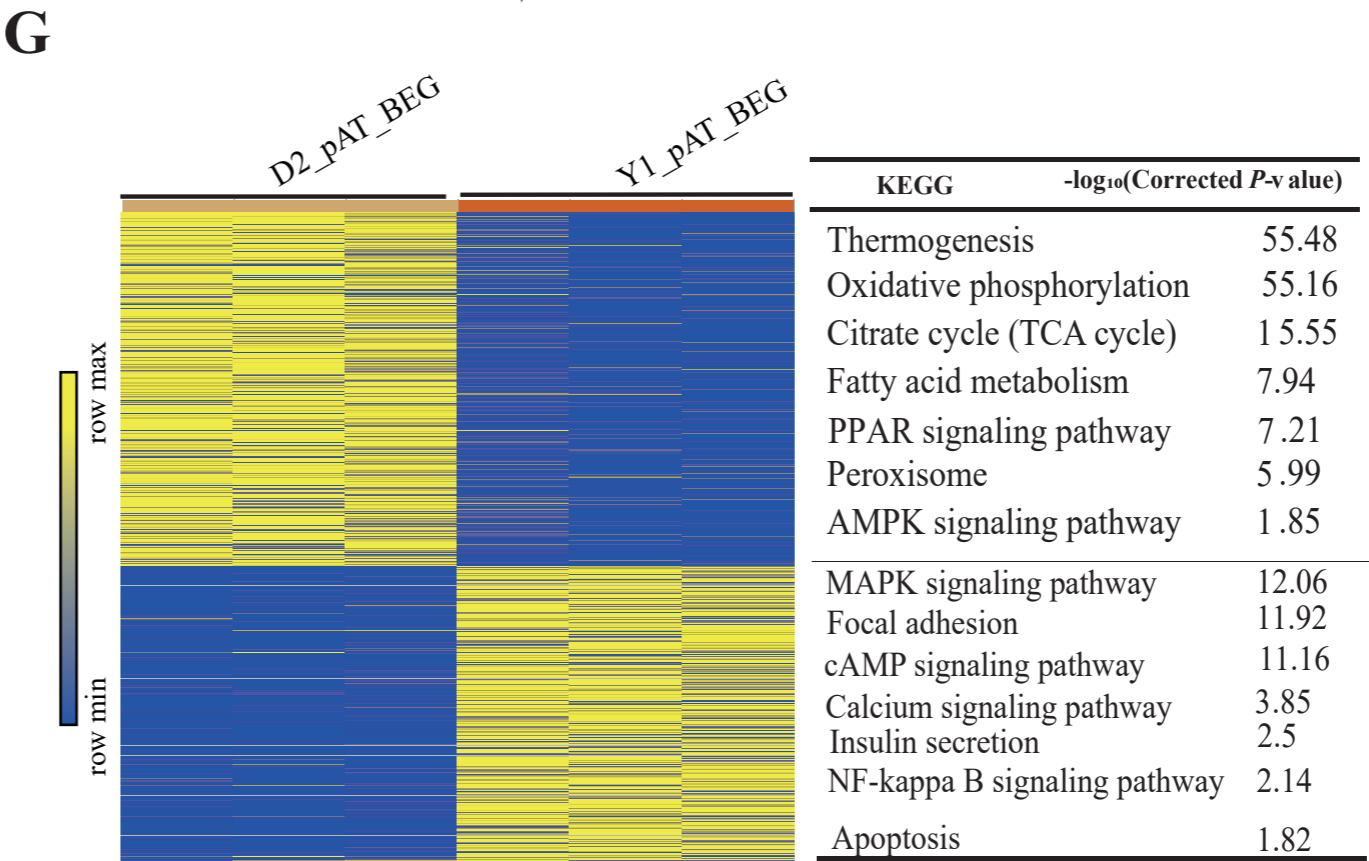

Supplement: Supplementary file 2 — Additional file 2: Fig. S1 Transcriptional remodeling of the pAT and oAT in goats. (A and B) Representative pictures of goat pAT and oAT at different ages. (C) The number of adipocytes per region (n=5) in histological images of the pAT and oAT in goats. (D) Heatmap displaying k-means clustering of DEG in goats pAT and oAT. The pathways enriched for each cluster were labeled in the right panel. (E) Pathway analysis of DEG detected by comparing the any two samples. (F) Pathway analysis of DEG detected by comparing the pAT and oAT at every age. (G) Heatmap displaying k-means clustering of DEG in big-eared goat pAT. The pathways enriched for each cluster were labeled in the right panel. **P <0.05, ***P <0.01 by one-way ANOVA followed by Bonferroni's multiple comparisons. pAT, perirenal adipose tissue; oAT, omental adipose tissue. [file 40104_2025_1157_MOESM2_ESM.pdf]

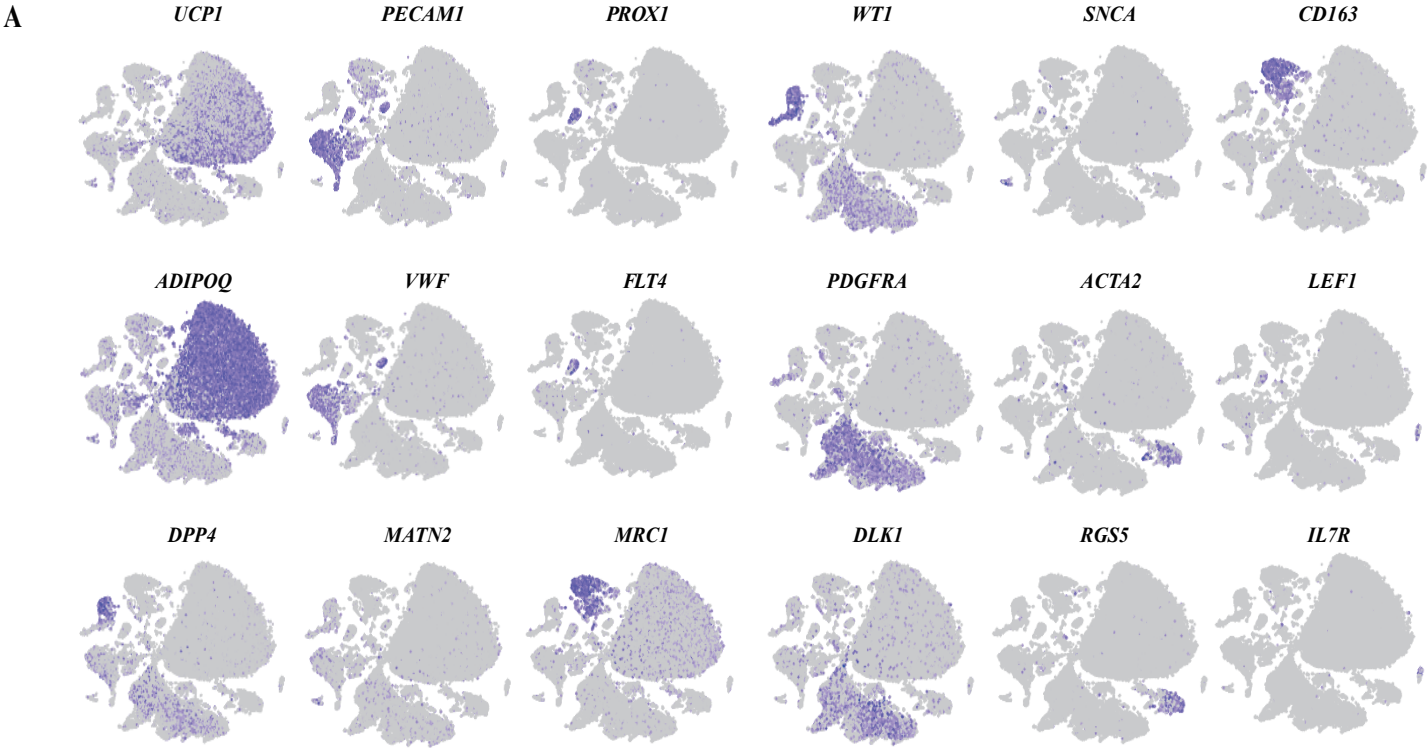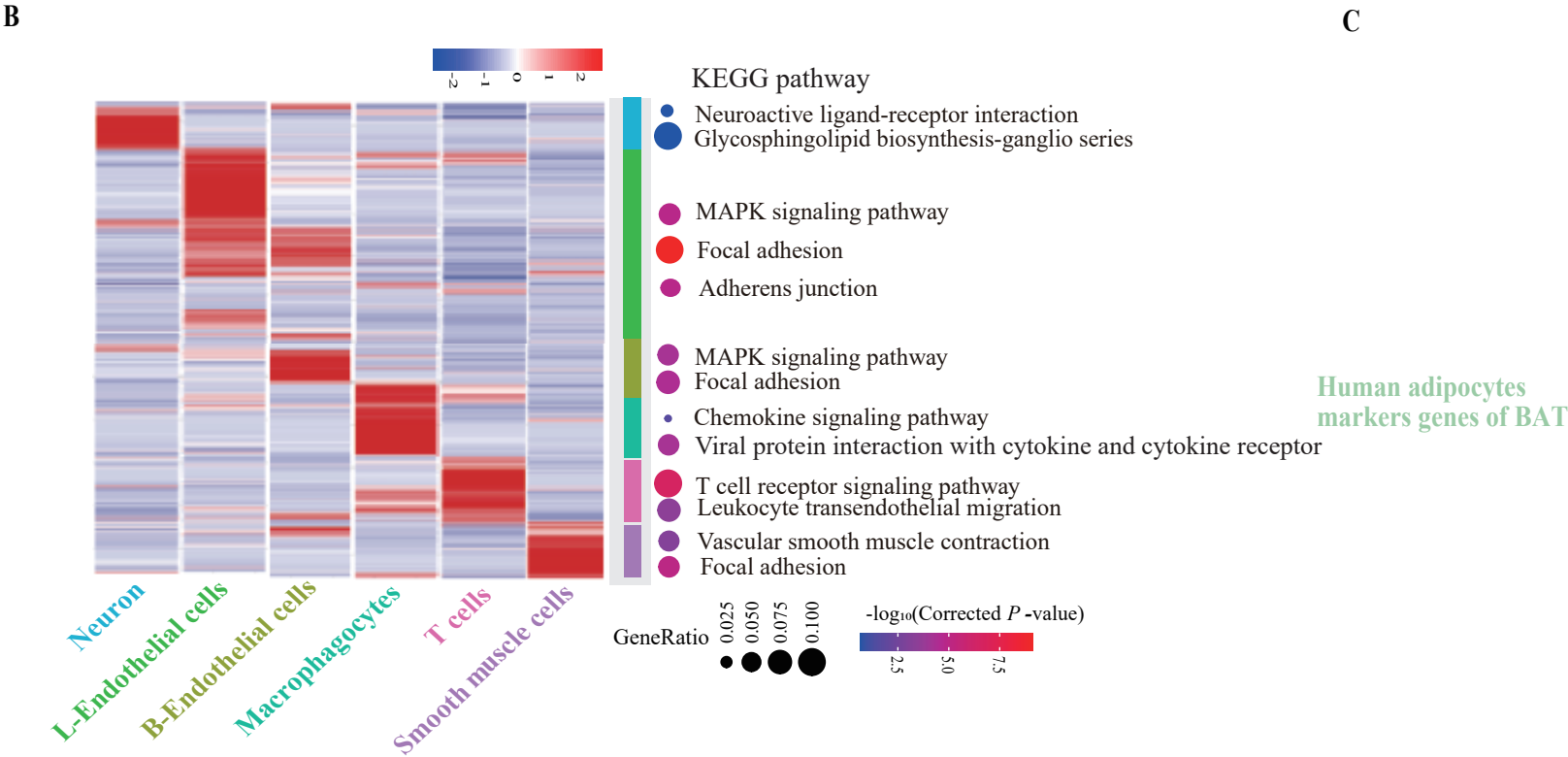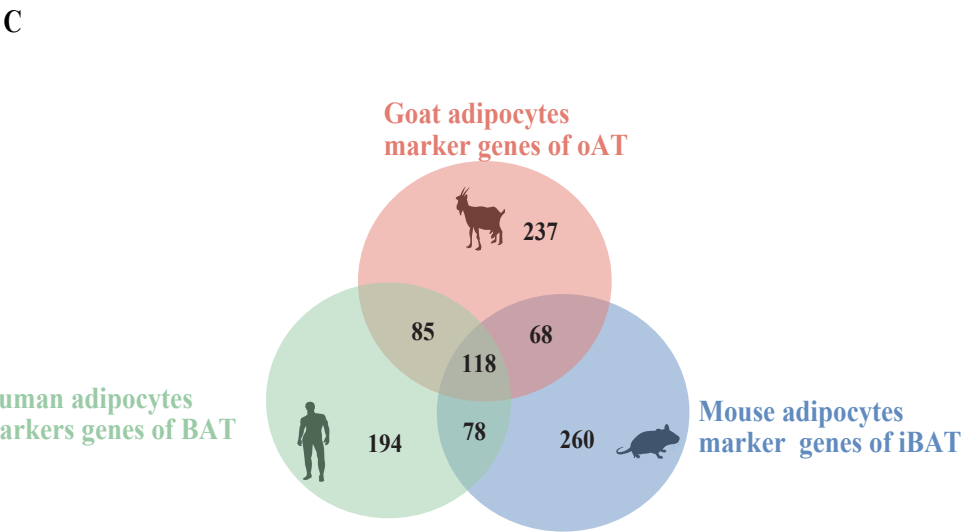

Supplement: Supplementary file 3 — Additional file 3: Fig. S2 Single-nucleus transcriptomic atlas of pAT and oAT in goats. (A) Feature plots showing the RNA expression levels of representative marker genes in 9 cell types of all 8 samples. (B) Heatmap for the RNA expression levels of the top 200 marker genes in 6 cell types (left panel). Dot plot showing the pathway enrichment of marker genes for each cell type (right panel). (C) Venn diagram illustrating overlapping marker genes with the highest 5% expression levels of thermogenic adipocytes in goat oAT, human BAT, and mouse iBAT. iBAT, interscapular BAT. [file 40104_2025_1157_MOESM3_ESM.pdf]

celltype   ● Adipocyte\_progenitors   ● Preadipocytes   ● Adipocytes

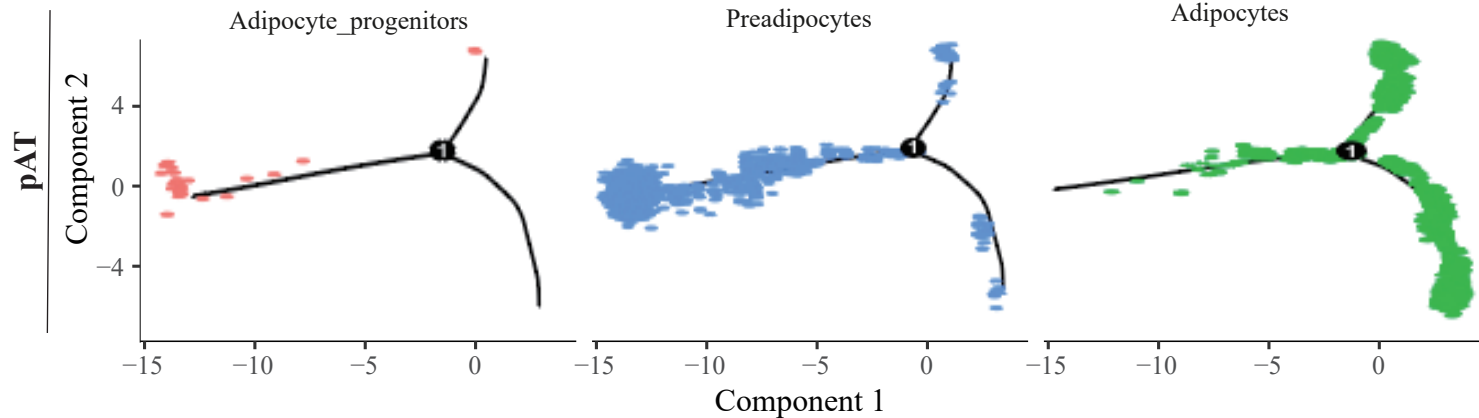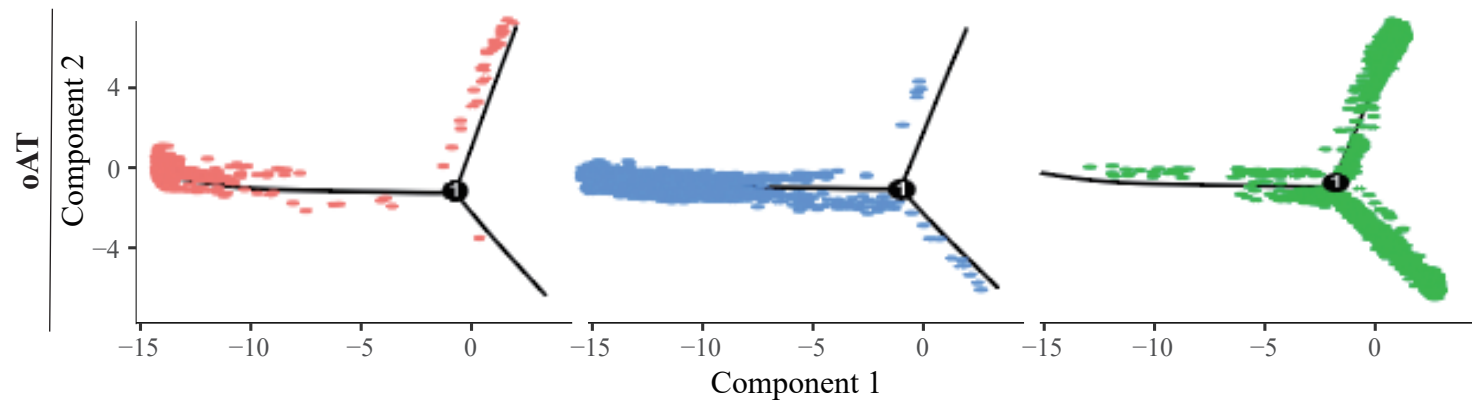

Supplement: Supplementary file 4 — Additional file 4: Fig. S3 Differentiation trajectories of the three cell types in pAT and oAT. pAT, perirenal adipose tissue; oAT, omental adipose tissue. [file 40104_2025_1157_MOESM4_ESM.pdf]

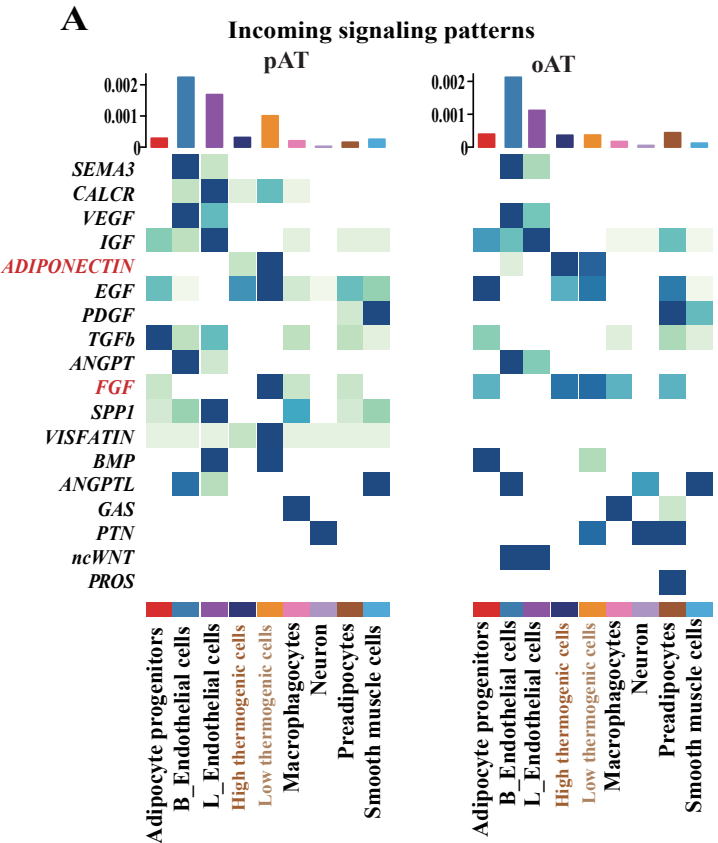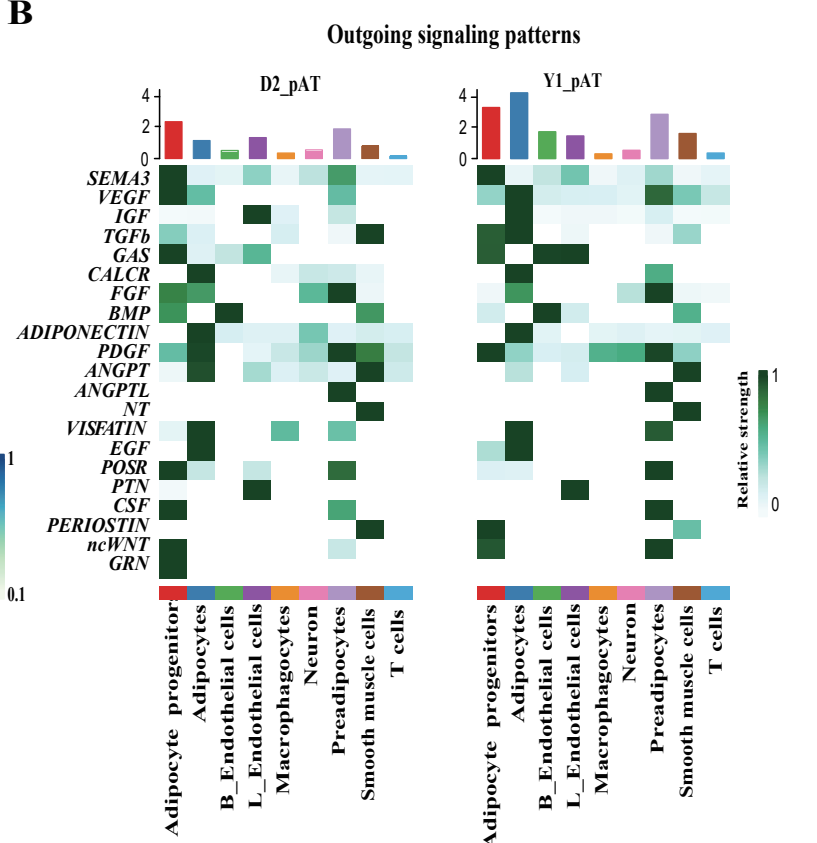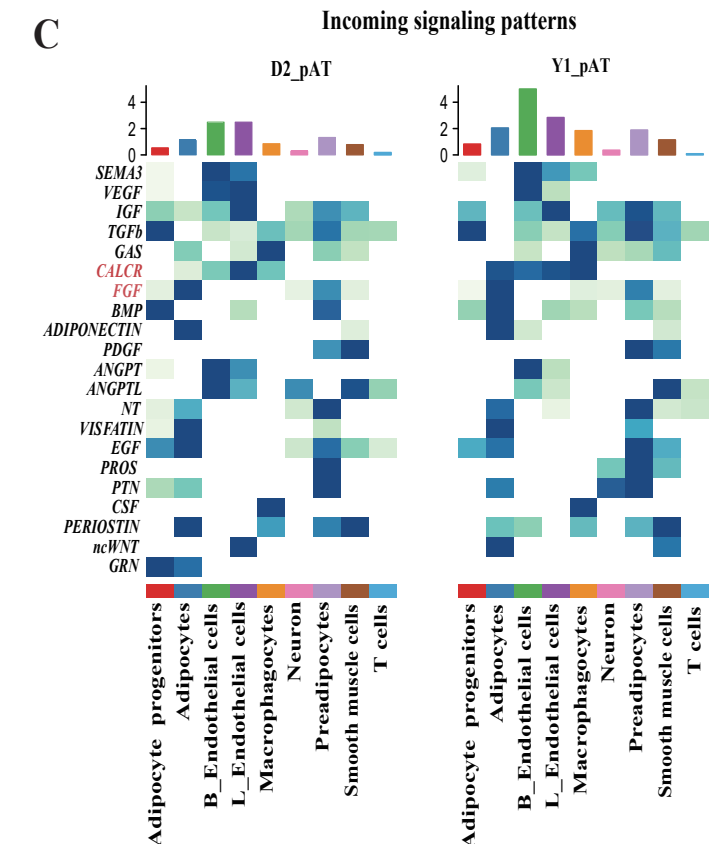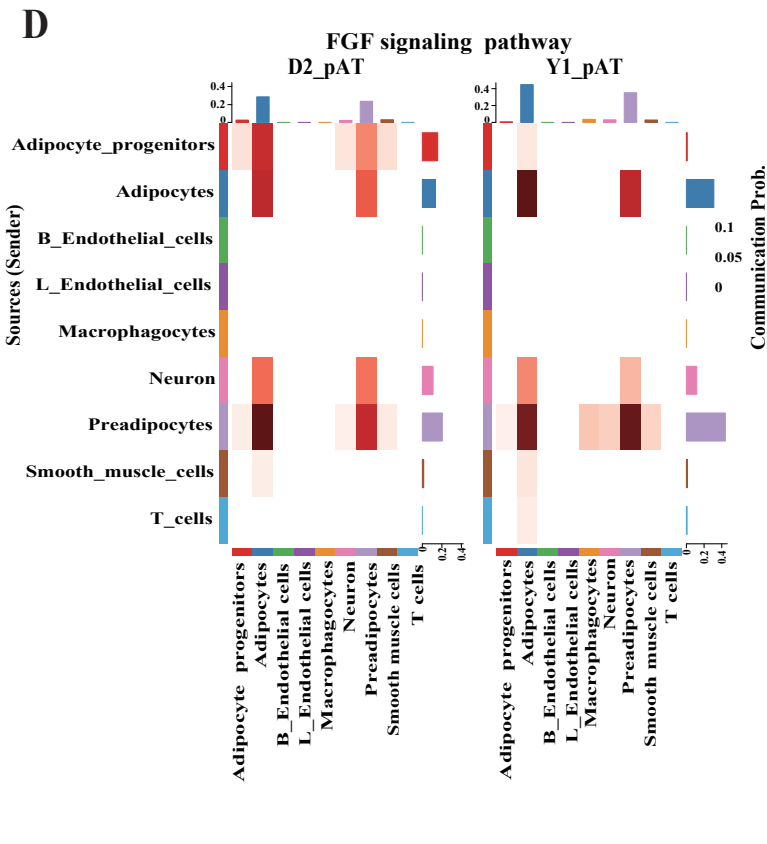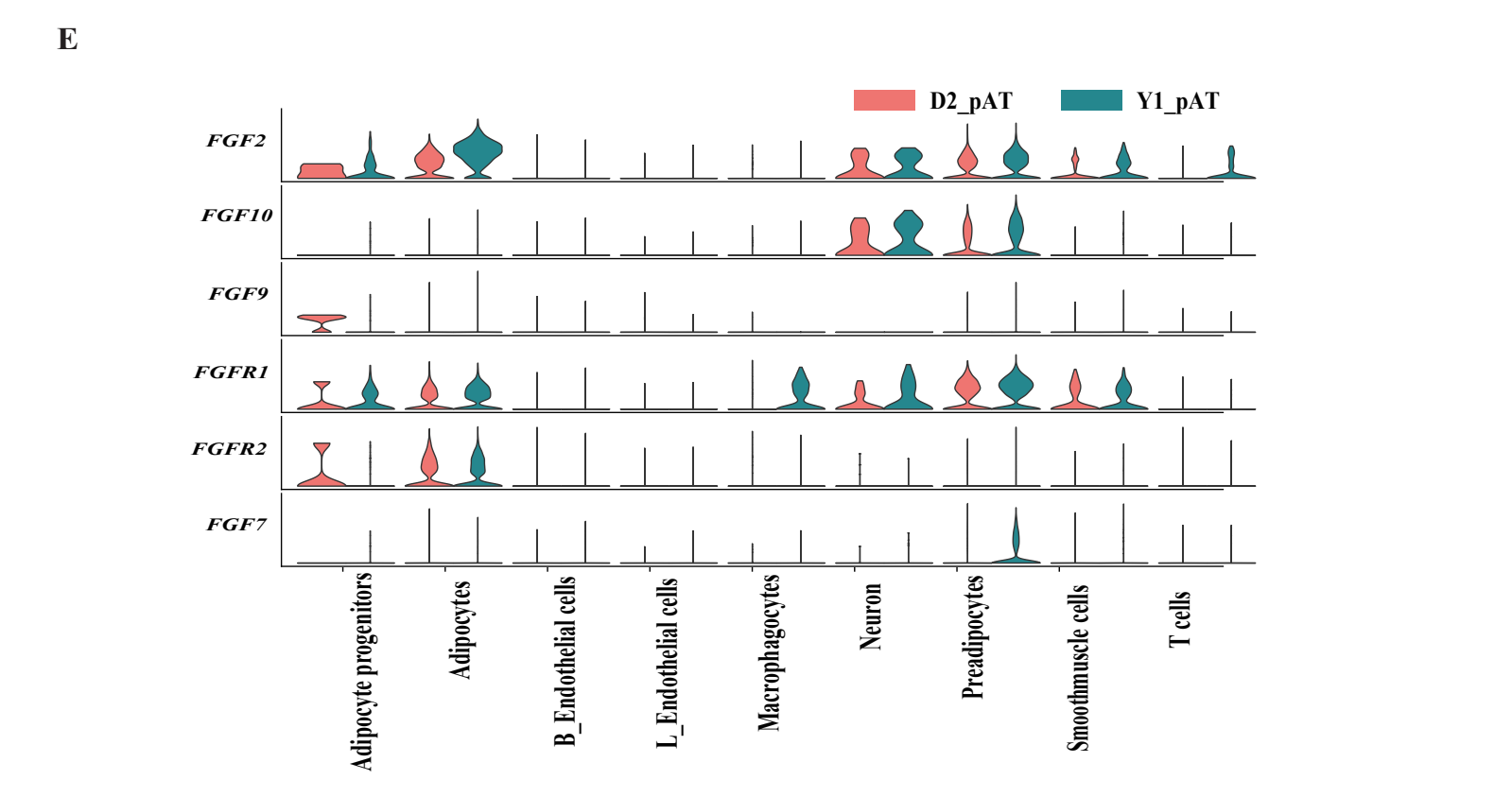

Supplement: Supplementary file 5 — Additional file 5: Fig. S4 The differential signaling in the cell-cell communication network analyzed by CellChat. (A) The incoming signaling patterns in pAT and oAT. (B) The snRNA-seq expression distribution of ligand and receptor in FGF signaling in pAT and oAT. (C and D) The outgoing (A) and incoming (B) signaling patterns in pAT and oAT. (E) The inferred FGF signaling network in D2 and Y1 pAT of big-eared goats. (F) The snRNA-seq expression distribution of ligand and receptor in FGF signaling in D2 and Y1 pAT of big-eared goats. pAT, perirenal adipose tissue; oAT, omental adipose tissue. [file 40104_2025_1157_MOESM5_ESM.pdf]
